# Insights into the roles and pathomechanisms of ceramide and sphigosine-1-phosphate in nonalcoholic fatty liver disease

**DOI:** 10.7150/ijbs.78525

**Published:** 2023-01-01

**Authors:** Cheng Zhu, Qian Huai, Xu Zhang, Hanren Dai, Xiaolei Li, Hua Wang

**Affiliations:** 1 Department of Oncology, the First Affiliated Hospital of Anhui Medical University, Hefei, Anhui, China.; 2 Inflammation and Immune Mediated Diseases Laboratory of Anhui Province, Anhui Medical University, Hefei, Anhui, China.

**Keywords:** Ceramides, NAFLD, NASH, lipid metabolism, therapeutics.

## Abstract

Non-alcoholic fatty liver disease (NAFLD), as one of the main causes of chronic liver disease worldwide, encompasses a spectrum of liver conditions that are not caused by other etiology, such as overt alcohol consumption, from simple steatosis to more aggressive non-alcoholic steatohepatitis (NASH) that involves liver inflammation and fibrosis, and to the lethal cirrhosis that may result in liver cancer and liver failure. The molecular mechanisms governing the transition from steatosis to NASH remain not fully understood, but the hepatic lipidome is extensively altered in the setting of steatosis and steatohepatitis, which also correlate with disease progression. With the tremendous advancement in the field of lipidomics in last two decades, a better understanding of the specific role of sphingolipids in fatty liver disease has taken shape. Among the numerous lipid subtypes that accumulate, ceramides are particularly impactful. On the one hand, excessive ceramides deposition in the liver cause hepatic steatosis. On the other hand, ceramides as lipotoxic lipid have significant effects on hepatic inflammation, apoptosis and insulin resistance that contribute to NAFLD. In this review, we summarize and evaluate current understanding of the multiple roles of ceramides in the onset of fatty liver disease and the pathogenic mechanisms underlying their effects, and we also discuss recent advances and challenges in pharmacological interventions targeting ceramide metabolism for the treatment of NAFLD.

## Introduction

NAFLD is the most common chronic liver disease, paralleling the worldwide increase of obesity 1, 2. NAFLD is a term that encompasses a group of pathologies ranging simple steatosis, NASH and cirrhosis. Fatty liver, or steatosis, is a disorder of deregulated lipid metabolism and is manifested by the excessive accumulation of lipid droplets in the cytosol of hepatocytes leading to toxicity. Hepatic damage is thought to be the main driving event behind the progression of NAFLD, causing injured or dying hepatocytes to release proinflammatory factors. Subsequent activation and infiltration of immune cells lead to inflammation and activation of hepatic stellate cells leads to fibrosis, furthering NAFLD progression 3-5.

NAFLD is also a lipotoxic disease characterized by insulin resistance, oxidative stress, adipokine secretion by adipocytes, endotoxins released by gut microbiota, and endoplasmic reticulum stress 6, 7. For many years, the most accepted explanation for NAFLD progression is the two-hit hypothesis. According to this hypothesis, the first hit is represented by lipid accumulation in the liver in the presence of metabolic alterations, such as high-fat diet, obesity and insulin resistance. Excess of lipids would then increase liver vulnerability to different factors (second-hits), which might be responsible for the progression from NAFLD to NASH 8, 9. The two-hit hypothesis is now obsolete, as it is inadequate to explain the several molecular and metabolic changes that take place in NAFLD. The multiple hit hypothesis considers multiple insults acting together on genetically predisposed subjects to induce NAFLD and provides a more accurate explanation of NAFLD pathogenesis. The multiple hit hypothesis refers to a perfect storm of multiple cells working simultaneously and interacting with each other during the development of NAFLD, leading to increased liver inflammation and liver fibrosis 8, 10. Although the deposition of large amounts of lipids in the liver and elevated circulating lipid levels are considered early events in NAFLD, it is less associated with subsequent progression of NAFLD. While simple steatosis follows a relatively benign clinical course, in some cases it may progress to NASH characterized with cell death and chronic inflammation, the level of ceramides and complex sphingolipids with ceramides as precursor are changed evidently in the liver, which may be one of the important factors for the progression of NAFLD 11, 12. Although a large number of studies have made much progress for understanding of the mechanisms involved in the complex pathophysiology of NAFLD, there are no current FDA-approved medications for NAFLD. Lifestyle modifications of weight loss (5%-10%) through methods, such as hypocaloric diets, aerobic exercise, and resistance training have been strongly advocated 13-15. Thus, there is a crucial need to identify multiple targets in NAFLD pathogenesis for the development of diagnostic markers and targeted therapeutics.

Sphingolipids comprise a highly dynamic, diverse, and complex class of bioactive molecules that act as both structural components of cellular membranes and signaling molecules in mammalian cells. Ceramides are a class of bioactive sphingolipids consisting of a fatty acid chain and are also considered as the central molecules in sphingolipid metabolism. Besides being indispensable components of cell membranes, ceramides act as signaling molecules to coordinate various cellular functions, such as cell proliferation, apoptosis, senescence as well as stress response in health and disease 16-18. Ceramides can be mainly synthesized through three pathways, including *de novo* pathway, sphingomyelinase pathway and salvage pathway 19-21. The bioactive sphingolipid metabolites ceramide and sphingosine-1-phosphate (S1P) are a recent addition to the lipids accumulated in obesity and have emerged as important molecular players in various metabolic syndromes, such as fatty liver disease, and insulin resistance 22, 23. Ceramide has been shown to accumulate in parallel to neutral lipids in livers of obese people and animals. Evidence has shown convincingly that sphingolipids, ceramide in particular, contribute to key steps in NAFLD onset, including the disruption of insulin sensitivity and mitochondrial metabolism, the metabolic derangement, and the stimulation of cell death 24, 25. In this review, we provide an overview of the pathogenic mechanisms by which ceramides mediate the pathogenesis and progression of NAFLD and we also discuss potential therapeutic modalities targeting ceramide metabolism for the treatment of NAFLD.

### Lipids accumulation in NAFLD

Lipid metabolism disorders are the primary causes for the occurrence and progression of fatty liver diseases. NAFLD is a condition of fat accumulation in the liver in combination with metabolic dysfunction in the form of overweight or obesity and insulin resistance. Hepatic steatosis is classified into various degrees based on lipid percentage in hepatocytes, which can further induce organelle dysfunction, cell injury, hepatocyte ballooning, apoptosis, and other pathophysiological changes that promote disease progression in NAFLD 26. The liver controls the metabolism of fatty acids through several molecular mechanisms, including the uptake and export of fatty acids, de novo fatty acid generation, and fatty acid oxidation. When the balance between these pathways is altered, hepatic lipid accumulation commences, and long-term activation of inflammatory and fibrotic pathways can progress to worsen the liver disease 27.

Accumulation of lipids in hepatocytes is closely related to the interaction between liver and adipose tissue. The main lipid uptake for the liver is through lipolysis in adipose tissue, which releases fatty acids into the blood that are then taken up by the liver by membrane proteins called fatty acid transporters. The main features of adipose tissue dysfunction include enlarged or compressed adipocytes and chronic low-grade inflammation, which can lead to increased levels of lipolysis in adipocytes and increased free fatty acids production. Circulating free fatty acid can also be generated from the absorption of dietary. Several studies identified that the development of NAFLD is often associated with Western dietary patterns, particularly excessive dietary fat intake 28, 29. It is well known that circulating free fatty acid pool in an obese state is held accountable for the majority of liver lipids in NAFLD. Uptake of circulating free fatty acid by hepatocytes is largely dependent on both the concentration of plasma fatty acids and the capacities of membrane-bound fatty acid transport proteins (FATPs), as well as cluster of differentiation 36 (CD36) 30, 31. The significance of CD36 in the pathogenesis of NAFLD onset has been studied because modulation of its expression in the liver directly effects hepatic steatosis. A study exploring the role of CD36 in lipid metabolism revealed that localization of CD36 on the plasma membranes of hepatocytes and CD36 palmitoylation are markedly increased in the liver of mice with NASH, which promotes free fatty acid uptake by hepatocytes and inhibits fatty acid β-oxidation, leading to intracellular lipid accumulation and increased inflammatory response. Blockade of CD36 palmitoylation in mice with NAFLD decreased its localization on the hepatocellular plasma membrane and impaired its function as free fatty acid transporter, suggesting that CD36 or some of its functional regulators may be a promising therapeutic approach for the prevention and treatment of NAFLD 32. CD36 can also interact with insulin-induced gene-2 (INSIG2) to increase the level of SREBP1 synthesis, promote *de novo* synthesis of fatty acid and induce hepatic steatosis 33. Of the six mammalian FATP isoforms, FATP isoforms 2 and 5 are the major isoforms present in the liver. In a high-fat fed mouse model, knockout or knockdown of FATP2 and FATP5 reduces hepatocyte fatty acid uptake, the level of triglyceride deposition in the liver and resisted diet-induced obesity in mice, indicating a role of FATP-mediated lipid uptake as a facilitator of hepatic steatosis 34. Following uptake, hydrophobic fatty acids do not diffuse freely in the cytosol and must instead be shuttled between different organelles by specific fatty acid binding proteins (FABP). FABP1, originally referred to as liver-FABP or L-FABP, is almost exclusively expressed in the liver. L-FABP promotes the intracellular transport of long-chain fatty acids and regulates cellular fatty acid uptake and lipid metabolism processes. It has been demonstrated that L-FABP levels are significantly increased in patients with NAFLD and can promote disease progression and insulin resistance processes 35. Therefore, enhanced intracellular trafficking of fatty acids in the liver of NAFLD patients may be shunting harmful fatty acids to storage, thereby promoting steatosis.

One of the main sources of fatty acids is *de novo* lipogenesis, a pathway in which fatty acids are synthesized from non-lipid precursors. Aside from triglyceride synthesis from externally derived fatty acids, the liver can also synthesize fatty acids *de novo* from a range of carbon sourced such as glucose, amino acid, which are then diverted to lipid droplets for storage as triglyceride. Three essential enzymes, acetyl-CoA carboxylase (ACC), Fatty acid synthase (FAS), and stearoyl-CoA desaturase-1 (SCD1), regulate *de novo* lipogenesis in liver. Individuals with NAFLD have increased rates of *de novo* lipogenesis and this is a major factor contributing to increase lipid deposition. Consistent with these observations, the expression of ACC and FAS are increased in the liver of patients with NAFLD or NASH. In *ob/ob* mice fed a high-carbohydrate diet, transient genetic inhibition of ATP-citrate lyase (ACLY) reduces liver lipid content. By contrast, using *in vivo* isotope tracing, it was shown that liver-specific inhibition of ACLY in mice cannot suppress fructose-induced lipogenesis. Dietary fructose is converted to acetate by the gut microbiota, and this supplies lipogenic acetyl-CoA independently of ACLY. Depletion of the microbiota or silencing of hepatic acetate-CoA synthetase 2, which generates acetyl-CoA from acetate, potently suppresses the conversion of bolus fructose into hepatic acetyl-CoA and fatty acids 36. The exact mechanism that drives *de novo* lipogenesis in NAFLD remains unknown, but activation of SREBP-1c and ChREBP due to increased circulating insulin and glucose in the context of whole-body insulin resistance has been suggested as a central driver 37. While limited, the available data collectively indicate that failure to regulate *de novo* lipogenesis is a central feature of liver lipid accumulation in NAFLD patients. Increased *de novo* lipogenesis represents an important mechanism driving hepatic triglyceride accumulation in fatty liver disease.

Fatty acids can be oxidized by multiple pathways. In fact, hepatic lipid accumulation leads to a compensatory increased oxidation, which mainly involves mitochondria. Activation of PPARα that is expressed at high levels in liver induces the transcription of a range of genes involved in mitochondrial and extramitochondrial fatty acid oxidation, thereby reducing hepatic lipid levels, and both whole-body and hepatocyte-specific ablation of PPARα in mice lead to reduced transcription of hepatic genes related to mitochondrial β-oxidation. Notwithstanding the relationship between PPARα activity and hepatic lipid metabolism, studies of PPARα expression levels in patients with steatosis or NASH are conflicting, reporting unchanged, increased and decreased PPARα expression 38, 39. The current data on fatty acid oxidation in NAFLD are conflicting, but even in studies suggesting augmented oxidation of fatty acids appear inadequate in clearing the liver of lipids. fatty acid oxidation in dysfunctional mitochondria—a characteristic of NAFLD—produces excessive ROS may ultimately facilitate disease progression by inducing oxidative stress and inflammation 40.

Toxic lipids deposited in the liver can lead to endoplasmic reticulum stress, mitochondrial damage to mediate hepatocyte injury and inflammatory responses 28, 41. Several studies have reported that the ratio of unsaturated fatty acids to saturated fatty acids (SFA) in free fatty acids is closely related to the progression of NAFLD. The accumulation of unsaturated fatty acids has no significant effect on liver cell viability, but the hepatic accumulation of saturated fatty acids promotes apoptosis and induces liver damage 42. Triglyceride molecules represent the major form of storage and transport of fatty acids within cells and in the plasma. NAFLD is characterized by excess triglyceride accumulation within the liver. Whereas previously it was considered that excess triglyceride stores in the setting of NAFLD contributed to lipotoxicity, emerging concepts suggest that increased triglyceride storage and is instead protective against fatty acids-mediated hepatotoxicity. It has been shown that inhibition of triglyceride synthesis reduces hepatic steatosis, but increases oxidative stress, inflammation levels and liver injury 43. During NAFLD, specific lipids, such as palmitic acid, cholesterol and ceramides have a destructive effect on hepatocytes, and these lipotoxic substances can affect cellular function through various mechanisms, including endoplasmic reticulum stress, activation of death receptors and mitochondrial dysfunction 44-46. Cholesterol homeostasis is mainly controlled by the liver. A balance between the availability of free and esterified cholesterol is critically important for hepatocellular function. When the synthesis and uptake of cholesterol exceed its removal, free cholesterol accumulates in hepatocytes. The role of cholesterol accumulation in NAFLD is an emerging topic, because it can further contribute to the alteration of the cellular function status. Free cholesterol overload in hepatocytes leads to ER stress, mitochondrial dysfunction, toxic oxysterols, and cholesterol crystallization in lipid droplets, thereby inducing hepatic cell death and inflammation 47, 48. Statins inhibit the synthesis of lipid cholesterol and prevent NAFLD and its progression 49. Although storage of triglycerides in the lipid droplets is a classical feature of NAFLD throughout the disease progression, triglycerides are presumed inert lipids that do not directly cause liver damage. Like cholesterol, ceramides play pivotal roles in all stages of NAFLD development, and in turn, lowering ceramides ameliorates NAFLD pathologies at multiple level 50-52.

### Ceramides biosynthesis pathway

**Figure 1:**
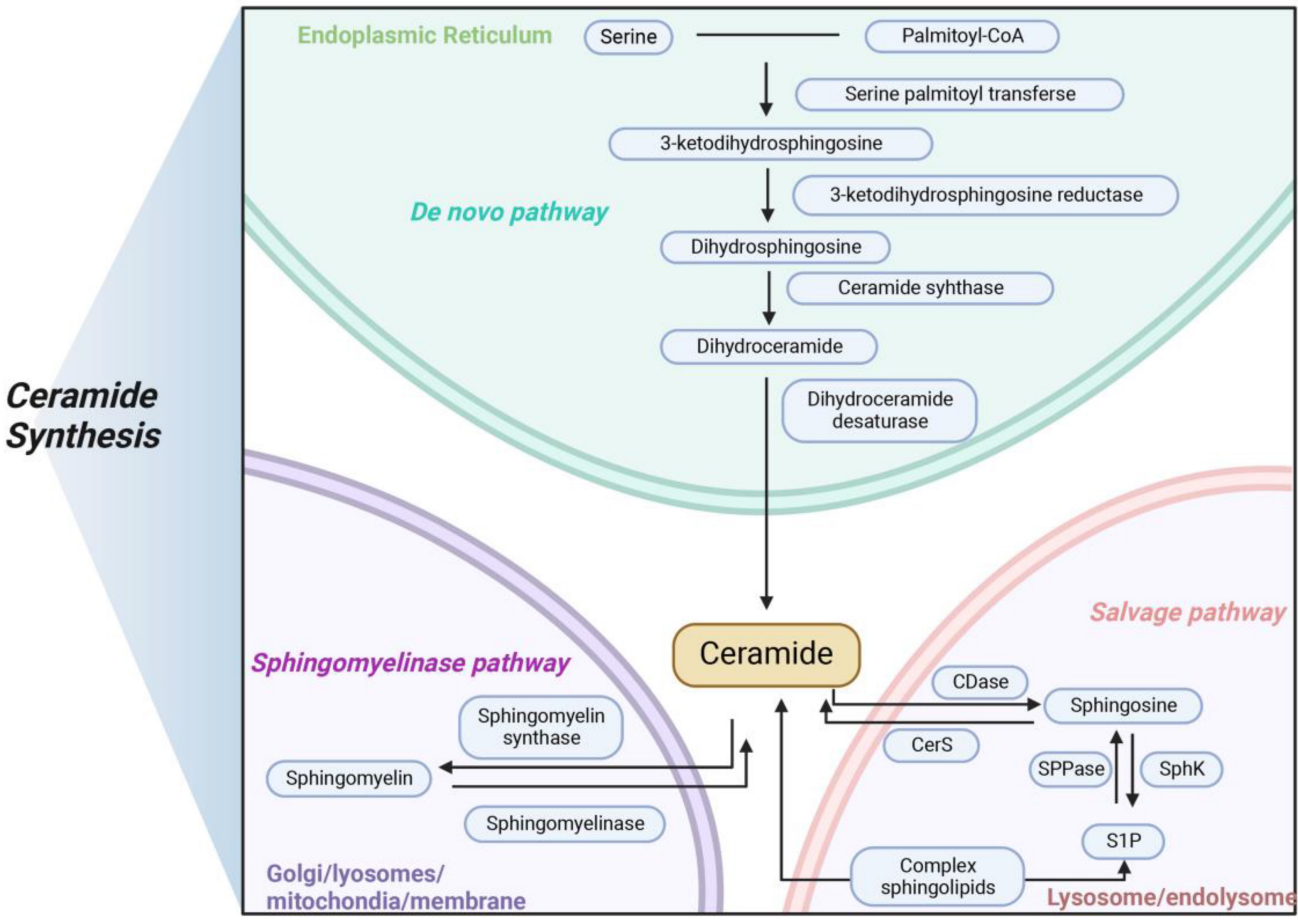
** Three main pathways of ceramides synthesis in mammals.** Ceramide is synthesized in three main ways, with* de novo* pathway occurring primarily in the endoplasmic reticulum, sphingomyelinase pathway occurring primarily in the lysosome, and salvage pathway occurring primarily in the Golgi apparatus. The three modes of synthesis work together to maintain the balance of ceramide metabolism *in vivo*.

Ceramides are a class of bioactive sphingolipids consisting of a fatty acid chain that form the backbone of more complex molecules, such as glucosylceramides and sphingomyelins. Ceramides with specific chain lengths have differing effects on both physiological and pathophysiological 53, 54. In addition to their structural role in the cell membrane, ceramides act as second messengers to coordinate various cellular processes, such as proliferation and apoptosis as well as stress response in health and disease 55, 56. Ceramide production takes place by three different pathways: the *de novo* synthesis pathway, the sphingomyelin pathway, and the salvage pathway (**Figure 1**).

Ceramide *de novo* synthesis begins in the endoplasmic reticulum with the condensation of L-serine and palmitoyl-CoA by serine palmitoyltransferase (SPT) to form 3-ketosphinganine which is then converted to sphinganine by 3-ketosphinganine reductase in an NADPH-dependent manner. Ceramide synthases (CerS) attach acyl-CoAs with different chain lengths to sphinganine to form dihydroceramides 57. Then, dihydroceramides are further dehydrogenated by dihydroceramide desaturase (DES) to form ceramide 58. After *de novo* synthesis in endoplasmic reticulum, ceramides are either taken to the Golgi by vesicular transport to be glycosylated into glucosylceramides or delivered by ceramide transport protein. When ceramides reach the Golgi, they are modified with a polar head to form sphingomyelin. Several of the enzymes required for de novo ceramide synthesis, including SPT and CerS. In mammals, there are large subunits of SPT, SPTLC1, 2 and 3, with SPTLC1 pairing with either SPTLC2 or SPTLC3 to form an active, heterodimeric complex. Two essential subunits, SPTLC1 and SPTLC2, are required for enzyme function. Other subunits, such as SPTLC3, alter substrate specificity, allowing alternative fatty or amino acids to be incorporated to produce less abundant sphingolipid species. In mammals, six CerS isoforms, called CerS1-CerS6, are expressed, with each generating dihydroceramides with specific acyl chain lengths, although redundancies exist between the different isoforms 59. These six CerS enzymes with a tissue-specific expression pattern, exhibit strict specificity in terms of the length of the fatty acid added to the sphingoid base and determine the fatty acid composition of sphingolipids in the cells 60, 61. Excepting CerS3, five of the six CerS enzymes are expressed in the intestinal mucosal cells, leading to the generation of a diverse ceramide pool with variable acyl chain lengths ranging from C14 to C34. CerS2 is the most abundant CerS in hepatocytes which explains the observations that its product C24:1 ceramide is the most abundant ceramide species in the mouse liver 62, 63.

The second important metabolic pathway for the acute production of ceramides is the sphingomyelinase pathway. Sphingolipids and ceramides can be interconverted by the action of sphingomyelinase and sphingomyelin synthase. The phosphodiester bond in the middle of sphingolipids can be hydrolyzed by sphingomyelinase to produce phosphorylcholine and ceramides. Sphingomyelinases can be divided into three types according to the optimal pH differences and subcellular localization: neutral sphingomyelinase, alkaline sphingomyelinase and acid sphingomyelinase 64, 65. While alkaline sphingomyelinase is mainly localized in the gastrointestinal tract and to some extent in the liver, neutral sphingomyelinase and acid sphingomyelinase are ubiquitous and account for the generation of ceramide in specific intracellular compartments, predominantly in the plasma membrane and lysosomes, respectively 66-68.

The salvage pathway of long chain sphingoid bases, leading to the re-generation of sphingolipids, has been estimated to contribute from 50% to 90% of sphingolipid biosynthesis. This pathway involves the degradation of glucosylceramide, sphingomyelin, and other complex sphingolipids, in acidic organelles such as the lysosome or late endosome, to form ceramides 69. A growing body of evidence is starting to point toward roles for ceramide generated through the salvage pathway in many biological responses, such as cell growth arrest, cell signaling and transport 21. Ceramides can be catabolized by acid ceramidases to form a sphingosine base and a free fatty acid chain, both of which are able to leave the lysosome, in contrast to ceramide which does not appear to leave the lysosome. The long chain sphingoid bases released from the lysosome may then re-enter pathways for synthesis of ceramide and/or S1P. In humans, five different ceramidases are known, which are differentially localized and are categorized according to their catalytic pH optimum: acid ceramidase (ASAH1), neutral ceramidase (ASAH2) and alkaline ceramidase (ACER1, ACER2, and ACER3). ASAH2 is highly expressed in the intestinal brush border membrane, ACER1 is often localized in the endoplasmic reticulum, ACER2 is mainly located in the Golgi complex, and ACER3 is widely expressed in the organism 70. In summary, these ceramidases have different cellular and substrate preferences, working together to regulate the body's ceramide levels.

### Liver ceramides contribution to NAFLD

**Figure 2:**
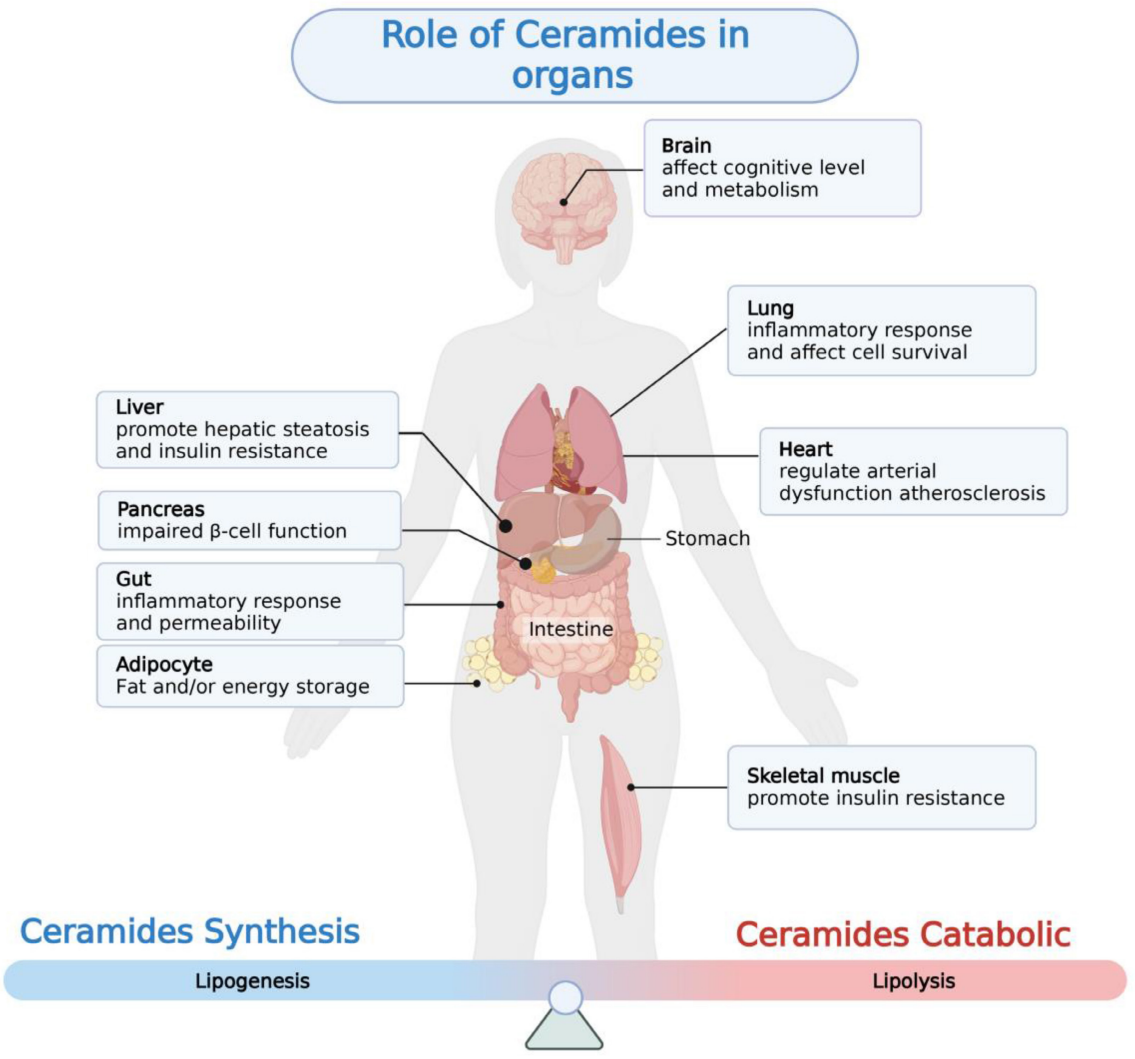
** Aberrant ceramides accumulation in a variety of tissues can lead to the development of a variety of metabolism-related diseases.** This figure depicts the pathological changes resulting from abnormal ceramide accumulation in different tissues.

**Figure 3:**
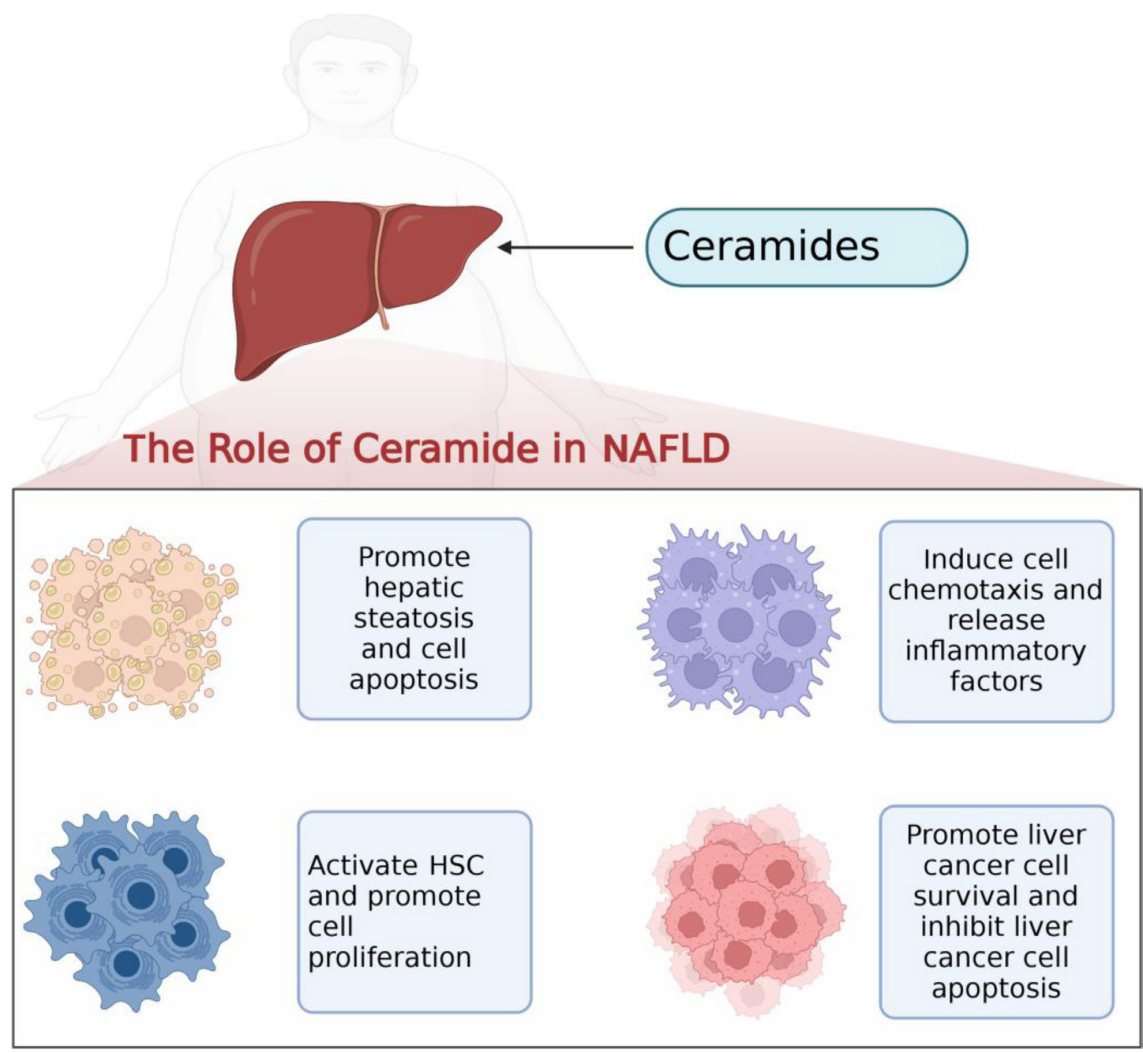
Ceramides on hepatocytes, hepatic stellate cells, macrophages and hepatocellular carcinoma cells in the progression of NAFLD.

With the rapid development of lipidomics approaches using mass spectrometry, cohort studies have revealed strong correlations between serum and liver ceramides levels and NAFLD pathologies in humans. Aberrant accumulation of ceramide in tissues is thought to be a negative regulator of glucose tolerance and lipid metabolism, which can modulate pathophysiological processes in a variety of diseases including insulin resistance, cardiovascular disease, coronary atherosclerosis, and diabetes 71-74 (**Figure 2**, **Table 1**). Evidence suggests that ceramide contributes to key steps in the onset and progression of NAFLD (**Figure 3**).

### Roles of ceramides in NAFLD

NAFLD represents a major chronic liver disease worldwide, characterized as progressive and developing in different stages. While simple steatosis follows a relatively benign clinical course, in some cases it may progress to NASH; a state characterized with cell death and chronic inflammation 75, 76. The onset and progression of NAFLD is associated with chronic elevation of ceramide content in hepatocytes. The emerging pathogenic roles of abnormal accumulation of ceramides in NAFLD, including inducing insulin resistance, promoting apoptosis, increasing endoplasmic reticulum stress and mitochondrial oxidative stress, and ultimately leading to steatosis, inflammation and fibrosis, have been demonstrated 25, 77, 78. One recent clinical study has reported that ceramides and other sphingolipid levels are significantly elevated in the liver of patients with NAFLD and NASH, and that ceramide levels are positively correlated with the degree of oxidative stress and inflammation in the liver 79. Similarly, Theodore et al. also found that ceramides were highly enriched in inflammatory steatosis hepatocytes by using in single-cell metabolomic analysis of the metabolic state of fatty acid-stimulated human hepatocyte lines, with the results validated *in vivo* and in line with previous reports 80. Based on these findings, ceramides have been shown to serve as biomarkers of NAFLD. The function of ceramides in NAFLD is also closely related to the length of the contained acyl chains. Wang et al. found that C16-ceramide synthesized by CerS5 and CerS6 promotes endoplasmic reticulum stress, leading to decreased liver function and metabolic imbalance in patients 81. However, CerS2-mediated synthesis of ultra-long chain ceramides protects against palmitic acid-induced lipotoxic endoplasmic reticulum stress in hepatocytes and attenuates insulin resistance levels and hepatic steatosis 82. In the presence of excess energy, excessive intake of saturated fatty acids increases plasma and intrahepatic ceramide levels more than polyunsaturated fatty acids 83-85. In the high-fat-fed mice model, the level of intrahepatic ceramides deposition in mice was significantly increased compared to the control group 86, implying that using different dietary patterns could regulate the level of ceramides. In addition, Dong et al. found that compared with control mice, hyperhomocysteinemia (HHcy) mice exhibited hepatic steatosis with a notable increase in ceramide-related metabolites and subsequent upregulation of ceramide synthesis genes. Furthermore, downregulation of ceramide levels through omega-3 polyunsaturated fatty acid supplementation ameliorates hepatic lipid accumulation, thus suggesting that ceramide may be a potential therapeutic target for the treatment of patients with hepatic steatosis 87. In addition, induction of liver-specific overexpression of acid ceramidase or deletion of CerS6 expression can also reduce ceramide levels, normalize lipid uptake and sphingolipid metabolism, and improve diet-induced hepatic steatosis 88.

### Ceramides induced hepatic insulin resistance

**Figure 4:**
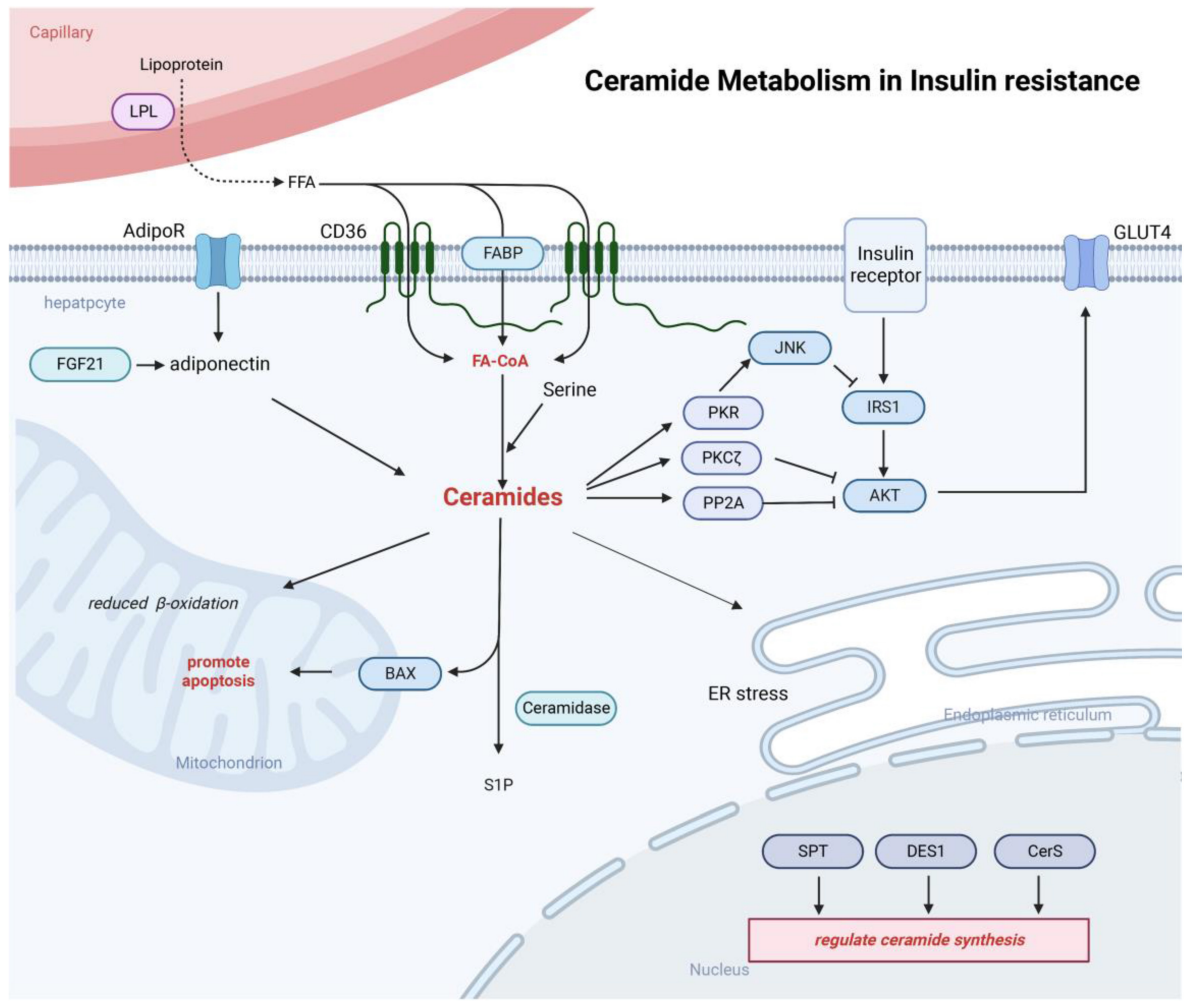
** Ceramides can be involved in the inhibition of insulin signaling.** Ceramide is able to inhibit AKT signaling pathway through both PP2A and PKCζ mechanisms and Inhibition of IRS-1 phosphorylation and the function GLUT4. Ceramide can also be involved in processes such as endoplasmic reticulum stress and mitochondrial dysfunction. Adiponectin released from adipose tissue can also be involved in the regulation of ceramide synthesis.

The pathophysiological mechanism of hepatic insulin resistance is very complex. On the one hand, accumulation of various lipids in the liver are key drivers of systemic and tissue-specific insulin resistance. On the other hand, tissue inflammation can induce insulin resistance 89, 90. Early investigations of ceramide-induced insulin resistance were performed primarily in skeletal muscle, and more recently, studies have confirmed that ceramides have been explored as an important mediators of insulin resistance in adipose and liver 20, 91-93 (**Figure 4**). Nevertheless, there are some contradictory findings on the role of ceramides in the induction of insulin resistance. In a study of obese non-diabetic patients, hepatic ceramide levels were not significantly correlated with insulin resistance 94. However, Hady et al. found a significant accumulation of intrahepatic ceramide in obese patients with diabetes, which was positively correlated with blood glucose levels, suggesting that ceramide may contribute to the induction of hepatic insulin resistance 95. In animal experiments, Xia et al. using overexpressing acid ceramidase mice found that the overexpressed acid ceramidase to consume excess ceramide in the liver, reduces intrahepatic ceramides deposition and improves hepatic insulin resistance 88. A recent study by Chaurasia et al. using ablation of the enzyme dihydroceramides desaturase 1 (DES1) from whole animals or tissue-specific deletion in the liver and/or adipose tissue found that inhibition of DES1 resolved hepatic steatosis and insulin resistance in mice caused by leptin deficiency or obesogenic diets, enhanced activation of AKT/PKB *in vivo*, and elicited a broad spectrum of metabolic benefits characterized by improvements in glucose and lipid handling. Meanwhile, ceramides also slow fatty acid turnover by increasing fatty acid storage in hepatocytes and reducing fat burning in adipose tissue. These findings highlight the role of ceramides in hepatic insulin resistance and also indicate that DES1 as a target that could be used to develop new treatments for various metabolic disorders 96.

In the liver, the ceramide mechanism of action on insulin resistance has not been fully elucidated. Several mechanisms have been proposed to explain how ceramides might induce insulin resistance, including: 1) ceramides impair insulin activation of AKT through two mechanisms: impaired AKT translocation via activation of PKCζ or activation of PP2A. Activation of PKCζ by ceramide can interact with AKT to induce AKT chelation into specific regions of the plasma membrane called caveolae and prevent AKT recruitment to the plasma membrane 97. Ceramides can also induce insulin resistance by activating PP2A, consequently hampering translocation of AKT from the cytoplasm to the plasma membrane. It has been shown that inhibition of PP2A in rats during AKT activation promotes hepatic insulin resistance, suggesting that insufficient activation of PP2A can also induce the development of insulin resistance 98; 2) In hepatocytes and muscle, ceramide uses an alternative pathway in modulation of insulin signaling that requires the phosphorylation and activation of PKR. Usually, insulin inhibits PKR activity by suppressing its phosphorylation. In liver cells, ceramide-activated PKR interferes with the insulin signaling pathway by phosphorylating IRS1 at Ser312 in an IKK/JNK-dependent manner, thus preventing the binding of IRS1 to PI3K 99; 3) a third pathway relates to the link between ceramides and GLUT4. Ceramide may inhibit glucose signaling by forming microstructural domains in phosphatidylcholine membranes, causing changes in plasma membrane fluidity, and avoiding GLUT4 fusion into the plasma membrane, thereby reducing glucose uptake and promoting insulin resistance. In addition, ceramide can also regulate the insulin resistance process by affecting free fatty acids, inflammatory cytokines, and glucocorticoids in various ways 100, 101. Adiponectin, a common adipocyte secretory factor, can enhance insulin sensitivity, increase ceramidase activity, and promote ceramide catabolism 102. In rodents, adiponectin could be activated by the metabolically active hormone fibroblast growth factor 21 (FGF-21), while diminishing accumulation of ceramides in obese animals. Moreover, FGF-21 lowers blood glucose levels and enhances insulin sensitivity in diabetic mice and diet-induced obese mice, only when adiponectin is functionally present 103.

Altering the acyl chain composition of ceramides may be a novel way of modulating insulin resistance and glucose metabolism. CerS1 and CerS6 induced ceramides of specific acyl chain lengths can lead to skeletal muscle insulin resistance, while ultra-long chain ceramides can improve the body's glucose metabolism levels 82, 104. One previous study has demonstrated that the level of C18:0 ceramide production in skeletal muscle was reduced in mice lacking CerS1, which effectively prevented obesity-induced insulin resistance 104. CerS2 null mice, which is unable to synthesize very long acyl chain (C22-C24) ceramides, exhibited glucose intolerance despite normal insulin secretion from the pancreas 19, 105.

### Role of ceramides in NASH

Pathological NASH develops when fat accumulation in hepatocytes is characterized by lobular inflammation and hepatocellular ballooning. The final stages of NASH are advanced fibrosis/cirrhosis, hepatocellular carcinoma, and liver failure. NAFLD has a strong association with obesity, type 2 diabetes, and hypertension, and is thus considered the hepatic manifestation of the metabolic syndrome. Inflammation, the central feature in the progression from simple steatosis to NASH, is subsequently triggered by metabolic overload and ensuing hepatocyte injury 106-108. Increasing intrahepatic ceramide levels in patients with NASH remains somewhat controversial. One study has indicated that hepatic ceramides are not only increased in insulin-resistant humans with NASH but also correlate with hepatic oxidative stress and inflammation, suggesting that intrahepatic ceramide levels may play a role during progression of simple steatosis to NASH in humans 109. However, another recent study has indicated that NAFLD is characterized by distinct changes in the liver lipidome with steatosis and the development of NASH does not result in further changes in the lipidome 79. Although ceramides accumulation in the liver and the progression of NASH are closely related, the underlying mechanisms by which ceramide involved in the different steps of NAFLD progression remain unclear. During the progression of NASH, saturated free fatty acids induce sublethal and lethal hepatocyte toxicity, in which endoplasmic reticulum stress is an important manifestation of sublethal hepatocyte toxicity. To cope with ER stress, the unfold protein response (UPR) is activated. UPR is a feedback regulatory system in which the endoplasmic reticulum detects the pressure of unfolded proteins in the lumen of the endoplasmic reticulum through intracellular signaling and is able to control the elimination of misfolded proteins in the endoplasmic reticulum to restore endoplasmic reticulum homeostasis. The unfolded protein mainly consists of three signaling branches containing inositol requiring enzyme-1 (IRE1), activating transcription factor 6 (ATF6), and protein kinase RNA-like endoplasmic reticulum kinase (PERK). *In vitro* model of palmitate-induced NASH, lipotoxic hepatocytes release extracellular vesicles (EV) containing ceramides mainly in an endoplasmic reticulum sensor-dependent manner to promote macrophage chemotaxis, and the use of IRE1 inhibitors or knockdown of its expression level reduces NASH inflammation as well as release of EVs. Activated IRE1A promotes the transcription of SPT through X-box binding protein 1 (XBP1), induces ceramides biosynthesis and EV release, thereby exacerbating the level of inflammation and the degree of liver injury *in vivo*. Meanwhile, the levels of XBP1, SPT and EV in the liver in NASH patients are significantly increased 52. Besides, innate immune abnormalities in the liver also play an important role in the pathogenic mechanisms of ceramides in NAFLD. Palmitic acid stimulates hepatocytes to produce S1P-rich EVs, and during this process, sphingosine kinases 1 and 2 are involved in regulating S1P enrichment levels in EVs, and then S1P exacerbates the level of inflammation in the liver by binding to S1P receptors on the surface of macrophages 110. Neutrophil aggregation is an early event in the NASH mouse model. In contrast to the beneficial effects during infection, neutrophils usually have deleterious effects on NASH through the production of ROS, cytokines, proteases and neutrophil extracellular traps (NETs). Neutrophil elastase (NE), another active protease secreted by neutrophils, has been shown to improve diets induced obesity and insulin resistance in mice. NE knockout improved western diet induced obesity, serum lipid disorders, steatosis and liver inflammation in NASH mice, which was possibly attributed to the potential of NE in regulating hepatic ceramides metabolism 111.

Ceramides have been reported as effectors in autophagy regulation, and are known to mediate apoptotic pathways. Thus, ceramides may play an important regulatory role in the autophagy function in the pathogenesis of NASH. However, the role of ceramides in autophagy is controversial. Ceramides were reported to either promote early autophagy and apoptosis or attenuate autophagy 112, 113. Noticeably, blockade of ceramide synthesis by myriocin could alleviate NASH severity, and improve the serum transaminases, triglycerides, cholesterol, liver pathology and fatty acid metabolism. Additionally, myriocin could also reverse the impaired autophagy function, indicating an important regulatory role of ceramides in autophagy function in the pathogenesis of NASH 114. Acid sphingomyelinase, a specific mechanism of ceramide generation, is required for the activation of key pathways that regulate hepatic steatosis and fibrosis, including endoplasmic reticulum stress and autophagy, and targeting acid sphingomyelinase can increase methionine cycling and phosphatidylcholine metabolism to slow the progression of NASH 115. Unlike the short-chain ceramides described above that exacerbate NASH, the available evidence suggests that long-chain ceramides promote hepatocyte survival. Wang et al. found that ACER3 is upregulated in livers of patients with NASH and in mouse livers with NASH induced by a palmitate-enriched western diet (PEWD). Knocking out Acer3 was found to augment PEWD-induced elevation of C18:1-ceramide and alleviate early inflammation and fibrosis but not steatosis in mouse livers with NASH, suggesting that targeting ACER3 represents a novel approach to prevention and intervention of NASH 116. Another study has shown that treatment with exogenous C24-ceramide also inhibited lipid synthesis and reduced intrahepatic lipid accumulation in a high-fat mouse model 117. Overall, most experiments have confirmed that excessive deposition of ceramides in the liver can promote NASH progression, exacerbate the level of liver inflammation, and promote oxidative stress and liver injury. However, due to the complexity of ceramide function, the exact mechanisms of its role in the different steps of NAFLD progression require further investigation.

### Role of ceramides in liver fibrosis

Liver fibrosis, which is manifested as excessive accumulation of extracellular matrix (ECM) proteins, is a common sequela to diverse chronic liver insults. Liver fibrosis is characterized by activation and proliferation of hepatic stellate cell (HSC), which are the major source of matrix-producing myofibroblasts 118. Compared to the roles in steatosis and liver inflammation, the role of ceramides in liver fibrosis is less clear. Liver fibrosis is accompanied by the hepatic accumulation of ceramide and sphingolipids, and excess ceramide regulates HSC activation and promotes hepatocyte apoptosis. However, the specific role of ceramides in liver fibrosis remains controversial. On the one hand, abnormal hepatic accumulation of ceramides has been observed in several mouse models of liver fibrosis, and as ceramide accumulates, cells undergo apoptosis and trigger the fibrotic events. For example, in mice on which fibrosis was induced with carbon tetrachloride treatment, the degree of fibrosis and inflammation in the liver is positively correlated with circulatory and intrahepatic ceramide levels 55. Activated HSCs produce ceramides via upregulation of key enzymes of ceramides synthesis pathway, and in turn, ceramides can activate HSCs and promote those cells to differentiate into profibrotic myofibroblasts, resulting in liver fibrosis 119. On the other hand, excess ceramides in HSCs attenuated liver fibrosis in mice. Pharmacologic inhibition or genetic knockout of acid ceramidase in HSC could inhibit YAP/TAZ signaling pathway activity, increase ceramides accumulation in HSCs and prevent liver fibrosis, implicating ceramide as a critical regulator of YAP/TAZ signaling and HSC activation and highlighting acid ceramidase as a therapeutic target for the treatment of fibrosis 120. Thus, ceramide is believed to be important in regulating apoptosis of hepatocytes. It is also a potential, major target for the treatment of NASH and fibrosis.

Dihydroceramide and S1P play also important roles in the progression of liver fibrosis. Sphingosine kinase is a key regulator of the dynamic ceramide/S1P rheostat balance. Ceramide usually inhibits proliferation and promotes apoptosis, while the further metabolite S1P stimulates growth and suppresses apoptosis. Repeated injections of purified hematopoietic stem cells markedly reduced liver fibrosis and improved liver function, which was increased by the co-administration of FTY720, a partial S1P receptor agonist, which enhanced the retention of hematopoietic stem cells inside the liver 121. In addition, increased dihydroceramide led to impairment of autophagic flux, and aggravated the progression of liver fibrosis. Due to the complex role of ceramide in liver fibrosis, targeting ceramide and its precursors could be one of the therapeutic approaches for liver fibrosis. Treatment with rapamycin recovered autophagic flux by regulating dihydroceramide could be a potential strategic approach for providing therapy for liver fibrosis 58. Chronic treatment with myriocin inhibited ceramide and lipid accumulation and improved liver fibrosis in the HFD-fed rat model, suggesting that ceramide synthesis could potentially be a target for the treatment of NAFLD 55.

### Role of ceramides in liver cancer

Metabolic alterations are a well-established hallmark of cancer. Deregulated lipid metabolism has been strongly correlated with the onset and progression of liver cancer. Ceramide not only regulates the differentiation, proliferation and apoptosis of tumor cells, but also mediates the inflammatory response in tumors 122, 123. Li et al. found that ceramide levels decreased but sphingomyelin level increased in hepatocellular carcinoma tissues by examining lipid expression in patients 124. But Bao M et al. found that ceramides, S1P, SM levels and species were significantly increased in hepatocellular carcinoma 125. These two paradoxical results may be due to changes in the signaling cascade of multiple cells during the development of liver cancer, resulting in dysregulation of enzyme expression related to ceramide metabolism. Ceramides are closely related to cell apoptosis and autophagy in tumors. In liver cancer, melatonin induced cellular autophagy by regulating the level of JNK phosphorylation, while specific acid sphingomyelinase-induced ceramide production participates in melatonin-mediated liver cancer cell death, suggesting that ceramide can modulate melatonin-induced autophagy and apoptosis in liver cancer cells 126. Systemic administration of nano-liposomal C6-ceramide to mice engrafted with liver cancer reduced tumor vascularization, induced tumor cell apoptosis, and ultimately blocked tumor growth 127. Ceramide can also regulate hepatocellular carcinoma cell migration. ACER2, one of the key enzymes that regulates ceramide hydrolysis, is overexpressed in hepatocellular carcinoma tissues and cell lines, and ACER2 knockdown resulted in decreased tumor cell growth and migration. Mechanically, ACER2 positively regulated the protein level of sphingomyelin phosphodiesterase-like 3B (SMPDL3B). Of note, ACER2/SMPDL3B promoted ceramide hydrolysis as well as S1P production that induced tumor cell growth and migration 128. As mentioned above, acid sphingomyelinase regulates the homeostasis of sphingolipids, including ceramides and S1P. Acid sphingomyelinase in the liver inhibits tumor growth through cytotoxic macrophage accumulation and tissue inhibitor of metalloproteinase 1 production by hepatic myofibroblasts in response to S1P 129.

The functional sphingolipid rheostat between ceramide and S1P has been well characterized in cancer therapy. Notably, the exogenous addition of acid sphingomyelinase or ceramide has been shown to augment the anti-tumor efficacy of sorafenib 130. Further data showed an upregulation of ceramide-induced cell death upon the stimulation of tumor cells with sorafenib and vorinostat 131. In addition, nano-liposome C6-ceramide (LipC6) in combination with anti-CTLA4 antibody represents a novel therapeutic approach with significant potential in activating anti-tumor immune response. In mice with liver cancer, injection of LipC6 alone can reduce the amount of TAM in the tumor, enhance the anti-tumor immune response and inhibit the growth of hepatocellular carcinoma, suggesting that LipC6 might increase the efficacy of immunotherapy 132. Tellingly, LipC6 in combination with anti‐CTLA4 antibody, but not anti‐PD‐1 antibody, significantly slowed tumor growth, enhanced tumor‐infiltrating CD8^+^ T cells, and suppressed tumor‐resident Tregs, representing a novel therapeutic approach with significant potential in activating anti-tumor immune response 133.

### Role of ceramide in organ-crosstalk

### Ceramides in adipose-liver crosstalk

**Figure 5:**
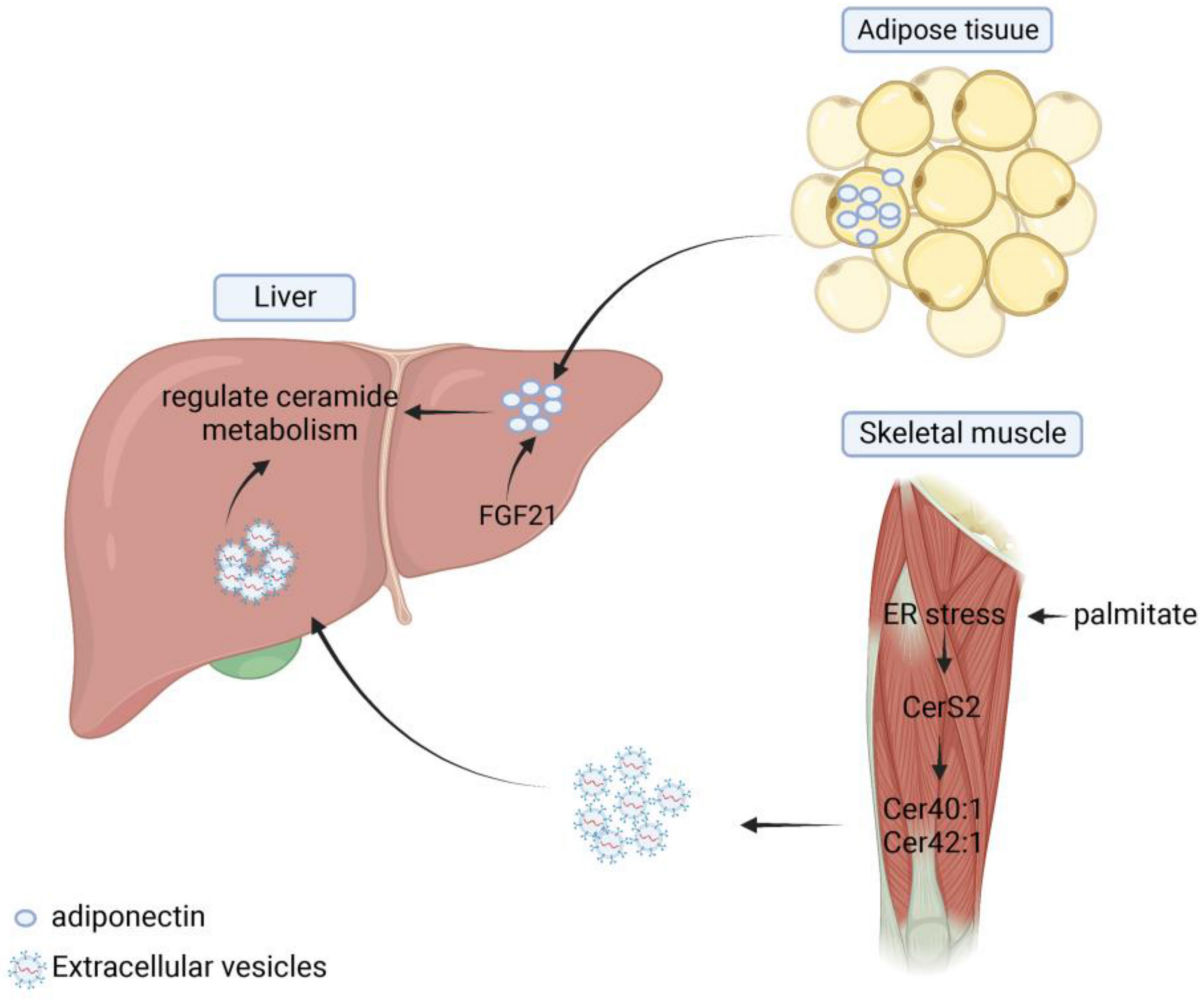
** The role of ceramides in organ crosstalk.** Adipose tissue can secrete adiponectin and thus regulate ceramide metabolism in the liver. Skeletal muscle can release extracellular vesicles containing Cer40:1 and Cer42:1 under endoplasmic reticulum stress conditions. These EVs can be absorbed by liver and regulate ceramide metabolism.

Metabolic syndromes, including NAFLD, can induce impaired adipose tissue function and release lipids to other organs, further leading to altered metabolic function, such as hepatic steatosis. Several studies have shown that there is organ-organ crosstalk in hepatic steatosis and that various lipids such as ceramides play a mediating role between different tissues and help maintain the homeostasis of the body's internal environment. In adipose tissue, inhibition of ceramide synthesis or promotion of ceramide hydrolysis can reduce the level of inflammation and hepatic steatosis within adipose tissue and improve systemic metabolic disturbances 134. Xia et al. showed that induction of acid ceramidase overexpression in adipose tissue reduced the level of hepatic steatosis and alleviated insulin resistance more rapidly and significantly than direct overexpression of the enzyme in the liver 88. Turpin's research group analyzed the differences in sphingolipid enrichment in different organs and tissues. The result showed that C16:0 was predominantly distributed in human adipose tissue, but that both C16:0 and CerS6 were significantly elevated in adipose and liver tissues in the obese population and in HFD-induced mouse models. In liver-specific CerS6 knockout mice, researchers found that mitochondrial β-oxidation and hepatic lipid metabolism levels were significantly increased in BAT, which not only improved energy supply but also reduced hepatic steatosis and insulin resistance 82. In addition, adiponectin secreted and produced in adipose tissue regulates glucose and lipid homeostasis by exerting pleiotropic effects on liver, pancreatic beta cells, kidney, heart and other tissues 102. Via adiponectin receptors, AdipoR1 and AdipoR2, adiponectin stimulates the deacylation of ceramide to produce S1P, thereby reducing ceramide accumulation and improving hepatic steatosis 101. FGF21 is a member of the fibroblast growth factor (FGF) superfamily, which increases the level of glucose and lipid utilization and regulates energy metabolism. William et al. suggested the existence of adiponectin-FGF21-ceramidase axis, the specific process of which is that FGF21 regulates the process of sphingolipid metabolism by inducing the level of lipocalin expression up and decreases ceramide levels, regulating sphingolipid metabolic processes (**Figure 5**) 103.

### Ceramides and gut-liver axis

**Figure 6:**
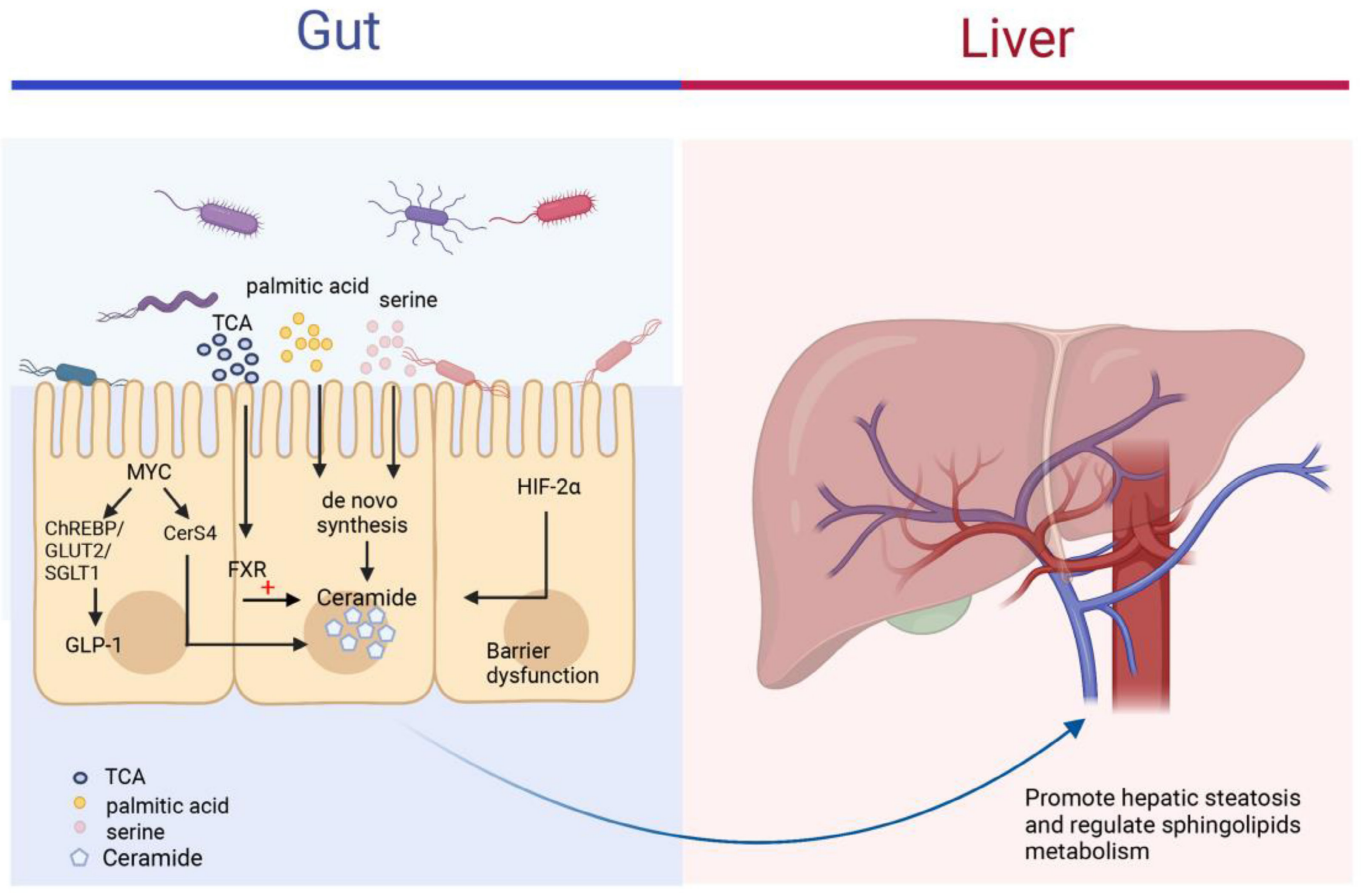
** The roles of ceramides in the gut-liver axis.** The figure mainly depicts the different mechanisms by which the different contents of the intestine can regulate ceramide synthesis. Gut microbiota can increase the risk of NAFLD through the synthesis of ceramide by uptake of palmitic acid and serine in the intestine and transfer to the liver via the portal vein to regulate sphingolipid metabolism in the liver. FXR and HIF-2α in the gut also regulate ceramide levels in the intestine and circulation.

The intestine plays an important role in the body's metabolism and physiology by transporting derived products to the liver with the help of the portal vein, which affects the pathophysiological functions of the liver 135, 136. When the microenvironment in the intestine is altered, the intestinal flora is involved in liver disease progression by activating various signaling pathways, such as FXR, hypoxia-inducible factor 2α, which directly or indirectly regulate ceramide synthesis and affect blood circulation and intrahepatic levels of fatty acids, ceramide and other sphingolipids (**Figure 6**) 137, 138. The level of ceramide synthesized within the intestinal flora varies somewhat under different dietary conditions. Di et al. gave germ-free mice, normal mice, and low germ mice fed linoleic or α-linolenic acid and analyzed the differences in circulating and intrahepatic fatty acid and sphingolipid deposition levels. The results showed that mouse intestinal microflora modulated circulating sphingolipid content and influenced the enrichment of Cer(d18:1/20:0), Cer(d18:1/24:1), and SM(d18:1/24:1) in the liver 139. In Asah2^-/-^ mice using a combination of dietary models of NAFLD/NASH, IgA secretion levels were altered in the intestine of Asah2^-/-^ mice compared to control mice, leading to increased synthesis of IgA-associated bacteria and their derivatives, decreased inhibition of sphingolipid synthesis, and improved hepatic inflammatory response 140. Different gut microbiota also differs in their effects on ceramide, lipids produced by Bacteroidetes can be transferred to the hepatic portal vein; Bacteroides thetaiotaomicron can increase ceramide levels in the liver and regulate sphingolipid metabolism 141. FXR is a nuclear receptor for bile acid specific response that regulates metabolic homeostasis in the intestine. Studies have shown that taurine-conjugated bile acids (TCA) can inhibit the process of small intestinal ceramide de novo synthesis and slow down the NAFLD process by inhibiting FXR 142. In intestine-specific FXR knockout mice, the expression of ceramide synthesis genes SMPD3/4, SPTLC2 and CERS4 were suppressed in the intestine, and ceramide synthesis levels were reduced, which contributed to the improvement of HFD-induced obesity, insulin resistance and hepatic steatosis 143, MYC has an important role as a proto-oncogene in cell proliferation, apoptosis and metabolic processes. The increased expression of intestinal MYC was observed in both obese people and mouse models, suggesting that intestinal MYC may be involved in the development of obesity-related metabolic syndrome. Luo et al. found that intestinal MYC not only regulates glucagon-like peptide-1 (GLP-1) secretion through ChREBP and GLUT2/SGLT1, but also regulates CerS4 expression and thus promotes ceramide synthesis. Intestinal-derived GLP-1 and ceramide enter the circulation as metabolic signals to the liver and various organs throughout the body, affecting the development of metabolic diseases such as obesity, insulin resistance, and fatty liver 144.

### Ceramides in other organ-liver crosstalk

Ceramides are mostly synthesized in the liver and affect sphingolipid metabolism in several organs throughout the body via the blood circulation. In cardiovascular disease, ceramides could cause endothelial dysfunction, affect the release of inflammatory factors and metalloproteinases, and promote atherosclerosis formation. Ceramides of different lengths have similar effects in cardiac insufficiency and liver diseases. High levels of C16:0 ceramide is detrimental to cardiac function, while very long chain ceramide C24:0 reduces the risk of cardiac events. Treatment with myriocin reduced high levels of ceramide-induced atherosclerosis and decreased hepatic lipid aggregation in mice 53. CYP7A1 in the liver is the rate-limiting enzyme in the bile acid synthesis pathway that can influence the progression of atherosclerosis. FGF15/19 binding to FGF receptor 4 could inhibit CYP7A1 expression in the liver and reduce circulating ceramide levels, which plays an important role in the prevention of obesity, insulin resistance and fatty liver disease 145. Except for cardiovascular, ceramides also play an important role in the metabolic interaction between skeletal muscle and the liver. Palmitate-induced cellular stress activates the *de novo* synthesis pathway of CerS2, inducing secretion of the long-chain ceramides Cer40:1 and Cer42:1. Ceramides are packaged into EV, secreted through the accumulation of dihydroceramide and induce UPR activation in naïve myotubes. Palmitate-induced endoplasmic reticulum stress in skeletal muscle can regulate systemic sphingolipid metabolism and mediate lipotoxic endoplasmic reticulum stress in adipose tissue and liver by activating CerS2 expression, promoting the accumulation of long-chain ceramides, and the release of EV containing ceramides 73. During obesity, skeletal muscle exhibits excess storage of various bioactive lipids, including diacylglycerol and ceramides, and by measuring changes in ceramide species in the skeletal muscle of dietary obese mice, researchers found increased levels of C18:0, which enhances skeletal muscle insulin resistance and accelerates the development of metabolic diseases 104.

### Ceramide and Sphingolipid metabolism as therapeutic target for NAFLD

Given the role of ceramides in the onset and pathogenesis of NAFLD, therapeutic approaches that target ceramide production could thus be of interest, including inhibition of ceramide synthesis or reduction of ceramide deposition levels. Next, we will further outline the drugs involved in ceramide as a therapeutic target for NAFLD (**Table 2**).

### Direct inhibition of ceramide synthesis process

Because of the closely correlation between ceramide deposition levels and NAFLD progression, inhibition of ceramide synthesis and reduction of ceramide key enzyme expression can improve NAFLD progression and stop the onset of a range of disease endpoints in NAFLD. A variety of ceramide synthase inhibitors have been identified that are able to target different CerS isoforms separately. Fumonisin B1, which was isolated from a fusariotoxin family associated with human esophageal cancer pathogenesis, was one of the first CerS inhibitors to be discovered and has a significant inhibitory effect on ceramide synthesis. However, fumonisin B1 is hepatotoxic, which leads to some limitations in its clinical application 146, 147. Turner et al. found that the selective CerS inhibitor P053 specifically inhibited CerS1 expression, thereby reducing C18:0 ceramide levels in cells and mouse skeletal muscle and improving insulin resistance 104. Another potential mechanism for reducing ceramide synthesis is the inhibition of the expression of the ceramide precursor which is dihydroceramide. Veeriah et al. found that administration of the potent HNF-4α agonist N-trans-caffeoyltyramine led to increased dihydroceramide levels by inhibiting dihydroceramide conversion to ceramides and promoted reversal of hepatic steatosis through a mechanism involving the stimulation of lipophagy by dihydroceramides 148. LipC6 as a non-toxic hydrophilic liposome carrier has been found to be clinically useful in the treatment of hepatocellular carcinoma. Recent studies have shown that LipC6 can reverse the imbalance of liver lipid metabolism induced by MCD feeding and reduce liver lipid deposition and inflammation levels by regulating AMPK/Nrf2 pathway and protecting antioxidant signaling pathway in a model of NAFLD/NASH 149.

### Indirect inhibition of ceramide production by inhibiting sphingolipid metabolism

SPT is a key enzyme in ceramide synthesis, and reduction of ceramide synthesis can be achieved by inhibiting SPT. Myriocin is a widely studied SPT inhibitor that irreversibly inhibits SPT expression and reduces ceramide levels, playing an important role in the treatment of diabetes, hepatic steatosis and atherosclerosis 150, 151. Interestingly, the novel immunomodulatory molecule FTY720, an analogue of myriocin, does not inhibit SPT, but is able to bind to S1P receptors, inhibit S1P lyase expression and participate in the regulation of sphingolipid metabolic homeostasis 152. In addition to the widely known myriocin, researchers have identified various other SPT inhibitors such as 3-(2-amino-ethyl)-5-[3-(4-butoxyl-phenyl)-propylidene] -thiazolidine-2, 4-dione (K145), cannabinoid-1- receptor (CB1R), etc. In NAFLD, K145 significantly improved fat accumulation in the liver of mice, and more importantly, it had no effect on lipid accumulation in normal liver 153, 154. CB1R inhibitors inhibit SPT activity while also suppressing CerS1 and CerS6 expression and reducing intrahepatic ceramide content 155.

### Suppression of free fatty acid (FFA) levels

Excessive deposition of FFA *in vivo* can promote ceramide synthesis in cells and tissues. Inhibition of FFA release may become one of the ways to regulate ceramide and mitigate the progression of metabolism-related diseases. Fenofibrate is a common lipid-regulating drug, and a recent study has shown that fenofibrate can slow the development of chronic inflammation by inhibiting hepatic ceramide synthase and reducing the accumulation of ceramides and lipids in the plasma and liver 156. In clinical studies, both saroglitazar and hepano were used for the treatment of hyperlipidemia and showed good inhibitory effects on ceramide. In addition, hepano has a superior modulating effect on sphingomyelins than saroglitazar, which has a significant hepatoprotective effect 157.

### Target to glucagon-like peptide-1 (GLP-1)

Liraglutide is a type of glucagon-like peptide-1 receptor agonist (GLP-1Ra) that acts directly on hepatocytes to alleviate hepatic steatosis 158. GLP-1Ra limit food intake and weight gain, additional beneficial properties in the context of obesity/insulin-resistance. Liraglutide could prevent accumulation of C16 and C24-ceramides/sphingomyelins species, inflammation and initiation of fibrosis in MCD-diet-fed mice liver, suggesting beneficial hepatic actions independent of weight loss and global hepatic steatosis 159. Other GLP-1Ra can also reduce ceramide synthesis, for example, exenatide can regulate lipid levels in type 2 diabetic patients based on improving glucose metabolism and insulin resistance, and significantly reduce circulating levels of sphingomyelin, ceramide, phosphatidylcholine and other lipids 160.

### Modification of dietary interventions and exercise

Both dietary interventions and changes in exercise patterns can reverse the elevation of ceramide in obesity, NAFLD, and other metabolic syndromes. Previous studies have confirmed that long-term adoption of Western diet, especially with high lipid intake, can promote the evolution of NAFLD, leading to elevated circulating and intrahepatic ceramide levels 85. In a cohort study of dietary interventions, Wang et al. found that plasma ceramide concentrations in patients were positively associated with cardiovascular disease risk, and that adoption of the Mediterranean diet attenuated elevated plasma levels of ceramide and delayed disease progression 161. Exercise or lifestyle changes can also reduce the accumulation of ceramides 162. After NASH patients received 1 year of lifestyle intervention to achieve weight loss, transcript levels of SPTLC2 and CERS1 in the liver were significantly reduced compared to controls without lifestyle intervention, and ceramide synthesis levels were reduced, reversing the NASH process 163. High resistance exercise and aerobic exercise can significantly improve liver and skeletal muscle insulin resistance, hyperlipidemia, obesity and other diseases related to the metabolic syndrome 164. Shepherd et al. also found a reduction in total ceramide levels in the muscles of obese men after continuous exercise training, with the most significant reduction in C18:0 ceramide levels in particular 165. In conclusion, the use of the right dietary interventions and exercise modalities can reduce ceramide accumulation in the body and thus improve the NAFLD process.

## Conclusion

Ceramide is considered the central molecule in sphingolipids metabolism and its modulation plays a key role in the development of several pathologies. Many *in vivo* and *in vitro* experiments have confirmed the closely relationship between abnormal ceramide metabolism and the pathogenesis of NAFLD. Due to the lack of effective drugs for the treatment of NAFLD, it is imperative to explore therapeutic modalities that may delay and reverse the disease. Given the important role of ceramides in orchestrating the progression of NAFLD, targeting ceramides offers a new therapy for the disease. Several therapeutic strategies focused on ceramide or other sphingolipid metabolizing enzymes that act as direct targets, including CerS, SPT, however, SPT is not a viable therapeutic target, owing to safety issues that result from the extreme diminution of all sphingolipids. In the future, treatments that focus on a more limited collection of harmful ceramide subseeds may represent a new strategy for therapeutic interventions. Currently, the vast majority of targeted drugs against ceramide are still in the preclinical stage and clinical trials are needed to confirm the effectiveness of the relevant drugs in humans. Due to the complexity of ceramide function, further study of the exact mechanisms of ceramide in disease development will facilitate the improvement of therapeutic strategies. Many questions remain to be explored at the present time, such as the specific functions of different chain-length ceramides remain unclear; whether the overall role of ceramides in liver fibrosis is pro- or anti-fibrotic; and whether the functions of ceramides are consistent in different organ tissues. Answering the above questions can help promote the possibility of ceramide-related drugs as a treatment for NAFLD. With the rapid development of single-cell metabolomics and transcriptomics, it has also increased our understanding of ceramide function in NAFLD and facilitated the search for potential therapeutic approaches targeting ceramide metabolism.

## Figures and Tables

**Table 1:** Summary of studies on ceramide modulation in different disease.

Diseases	Ceramide modulation	Reference Number
Alzheimer's disease	C18:1/18:0, C18:1/20:0↑	166
	Cer-C16:0, C18:0, C20:0, C24:0	167
	Cer-C16:0, C18:0, C20:0, C24:0	168
Multiple sclerosis	plasma: C16-Cer, C24:1-Cer, C16-GlcCer, C24:1-GlcCer ↑	169
	cerebrospinal fluid: C16:0, C24:0-Cer ↑	170
Cardiovascular disease	Ceramide ratio: Cer18:1\/C16:0、Cer18/C18:0 and Cer18.1\/C24:1↑	171
	Cer16:0\/C24:0 ↑or Cer24:0\/C16:0 ↓	172
Type 2 diabetic subjects	C16:0-Cer, C18:0-Cer, C22:0-Cer and C24:0-Cer ↑	173
	Cer-C18:0, C20:0, C22:0, DHCer-C22:0	174
NASH	dihydroceramides (16:0, 22:0, and 24:1) and lactosylceramides↑	109
Colorectal cancer	Cer-C16:0, C24:0, C24:1↑	175
Rheumatoid arthritis	Cer-C16:0, C22:0, C23:0, C24:1	176

**Table 2:** Drugs targeting ceramide and Sphingosine-1 Phosphate for NAFLD.

Types	Targets	Reference Number
P053	A selective inhibitor of ceramide synthase 1	104
Myriocin	The inhibitor of SPT	150, 177
CB1R	Inhibit the activator of SPT and expression of CerS1 and CerS6	155
Fenofibrates	lowering plasma triglycerides (TG) and ceramide	156
exenatide	The mimics of GLP-1	160
Liraglutide	The activator of GLP-1	159
K145	The inhibitor of SPHK2	153, 154
NCT	The activator of HNF-4α	178
lipofermata	The inhibitor of FATP2	179
hepano	ayurvedic polyherbal formulation	157
saroglitazar	a dual PPAR α/γ agonist	157
Fenretinide	Target DES and reduce the de novo synthesis of ceramide	180, 181
Fumonisin B1	The inhibitor of CerS	146, 147
Safingol	The inhibitor of SPHK1	181
LipC6	containing short chain C6-Ceramide	132

## Abbreviations

ACER1-3: alkaline ceramidase
ACLY: ATP-citrate lyase
ASAH1: acid ceramidase
ASAH2: neutral ceramidase
ASMase/ASM: acid sphingomyelinase
ATF6: activating transcription factor 6
CB1R: cannabinoid-1-receptor
CD36: fatty acid translocase
CerS: ceramide synthase
DES: dihydroceramide desaturase
EV: extracellular vesicles
FFA: free fatty acids
FABP: fatty acid binding protein
FATP: fatty acid transport proteins
GlcCer: glucosylceramide
GLP-1: glucagon-like peptide-1
HFD: High-fat diet
HHcy: Hyperhomocysteinemia
HSC: hepatic stellate cell
INSIG2: insulin-induced gene-2
IKK: IκB kinase
IRE1: inositol requiring enzyme-1
K145: 3-(2-amino-ethyl)-5-[3-(4-butoxyl-phenyl)-propylidene] -thiazolidine-2, 4-dione
MCD: methionine and choline-deficient
NAFLD: Non-alcoholic fatty liver disease
NASH: Non-alcoholic steatohepatitis
NET: neutrophil extracellular traps
NE: neutrophil elastase
PERK: protein kinase RNA-like endoplasmic reticulum kinase
PEWD: palmitate-enriched Western diet
PKA: protein kinase A
PKR: RNA-dependent protein kinase
sAC: soluble adenylyl cyclase SFA saturated fatty acids
S1P: Sphingosine 1-Phosphate
SM: sphingomyelin
SMPDL3B: sphingomyelin phosphodiesterase-like 3B
SPT: serine palmitoyltransferase
TCA: taurine-conjugated bile acids
UPR: unfold protein response
XBP1: X-box binding protein 1
